# Fabrication of Multifunctional Films Incorporating Purple Sweet Potato Anthocyanins and ZIF-8-NH_2_@Rt for Monitoring and Preservation of Pork Freshness

**DOI:** 10.3390/polym18141699

**Published:** 2026-07-10

**Authors:** Yangjie Huang, Haixia Wang, Yiyuan Zhang, Yuhang Liu

**Affiliations:** School of Materials Science and Chemical Engineering, Harbin University of Science and Technology, Harbin 150040, China; 19811733378@163.com (Y.H.); zhangyiyi2620@163.com (Y.Z.); rainhang1iu@163.com (Y.L.)

**Keywords:** pH-responsiveness, rutin, ZIF-8-NH_2_, guar gum, polyvinyl alcohol

## Abstract

Natural antioxidants are limited in food packaging due to poor stability and compatibility. A multifunctional film was successfully prepared via the incorporation of functional fillers into a guar gum/polyvinyl alcohol (GP) matrix. ZIF-8 was amino-functionalized to enhance rutin (Rt) loading, and the resulting ZIF-8-NH_2_@Rt was combined with purple sweet potato anthocyanins (PSPA) to fabricate a composite film for pork freshness monitoring. Compared with neat GP, the ZIF-8-NH_2_@Rt/PSPA/GP film showed a 30.6% increase in tensile strength and an elongation at break of 36.6%. The composite film also imparted exceptional UV-blocking capabilities, with light transmittance plummeting to 3.22% in the UVA region, 0.40% in the UVB region, and 47.53% within the visible light spectrum. The films displayed significant antioxidant properties, with DPPH and ABTS scavenging activities recorded at 73.27% and 67.64%, respectively. During pork storage, the film exhibited stable color changes. The G/R values corresponding to the limit of edibility were determined to be 0.78 and 0.67 for storage at 25 °C and 4 °C, respectively. The film was reusable for four cycles and extended the shelf life of pork by at least one day. These findings highlight the film’s considerable promise in the realm of smart food packaging and dynamic freshness tracing.

## 1. Introduction

The growing global demand for high-protein animal-derived foods, along with rising consumer concerns about food safety, has driven significant advancements in intelligent and active packaging technologies [1]. Protein-rich products, such as pork [2], poultry [3], and seafood [4,5], are particularly susceptible to spoilage caused by microbial growth and endogenous enzymatic activity. These degradation processes lead to the accumulation of total volatile basic nitrogen (TVB-N) mainly consisting of ammonia, dimethylamine, and trimethylamine causing detectable pH changes in the food matrix [6]. As a result, pH-responsive sensing materials play a crucial role in real-time, non-destructive monitoring of food freshness. Among these, natural pigments such as anthocyanins and curcumin stand out due to their biocompatibility, eco-friendliness, and low toxicity [7].

Anthocyanins, a class of water-soluble flavonoids, demonstrate distinct pH-dependent color transitions, rendering them ideal candidates for intelligent packaging applications [8]. Multiple botanical sources, such as purple sweet potato, mulberry [9], blueberry [10], red cabbage [11] and black carrot [12], have been employed to develop bioactive films capable of both freshness indication and shelf-life extension through antioxidant and antimicrobial activities. Notably, purple sweet potato anthocyanins (PSPA) are characterized by abundant acylated cyanidin and peonidin glycosides, endowing them with exceptional thermal stability, lightfastness, and a wide colorimetric response range [13]. In addition to anthocyanins, rutin (quercetin-3-rhamnosyl-glucoside), a flavanol prevalent in fruits and vegetables, exhibits strong antioxidant and antimicrobial activities [14]. Although classified as a polyphenol, rutin demonstrates poor water solubility and structural instability during processing [15].

Recent progress in active packaging has focused on incorporating naturally derived bioactive compounds with functional nanofillers to enhance preservation efficacy [16]. However, the practical application of PSPA and rutin is severely constrained by their susceptibility to degradation under environmental stressors, including UV radiation, heat, and humidity [17]. To tackle this instability, metal–organic frameworks (MOFs), notably aminated ZIF-8, have been utilized as innovative carriers. Their exceptionally high specific surface area and tunable pore architecture enable efficient immobilization and controlled release of labile bioactive compounds, thereby significantly enhancing their operational stability [18].

Herein, we report a novel multifunctional smart packaging film fabricated by incorporating rutin-loaded ZIF-8-NH_2_ (ZIF-8-NH_2_@Rt) and PSPA into a guar gum/polyvinyl alcohol (GP) blend matrix. This design aims to achieve a synergistic effect: the MOF carrier enhances the stability and bioavailability of rutin, while the coexistence of Rt and PSPA imparts robust antioxidant capacity and sensitive pH-responsive colorimetry. Furthermore, the integration of nanofillers reinforces the mechanical strength and moisture barrier properties of the composite films. The systematic evaluation of physicochemical characteristics, antioxidant capacity, and freshness-indicating performance reveals the potential of these films for sustainable intelligent food packaging applications.

## 2. Materials and Methods

### 2.1. Materials and Reagents

Guar gum (CAS: 9000-30-0, viscosity: 5000–5500 mPa·s) was obtained from Aladdin Chemical Co., Ltd. (Shanghai, China). Polyvinyl alcohol (Mw: 10, 1200, degree of hydrolysis: 99%) was supplied by Yousuo Chemical Technology Co., Ltd. (Linyi, China). PSPA was purchased from Xabcbiotech Co., Ltd. (Xianyang, China). Glycerol (99%), methanol, and ethanol were acquired from Fuyu Fine Chemical Co., Ltd. (Tianjin, China). Zinc nitrate hexahydrate (Zn (NO_3_)_2_·6H_2_O, 99%), 2-methylimidazole (98%), 2-aminobenzimidazole (98%), and Rt (95%, Mw = 610.52) were purchased from Acmec Biochemical Co., Ltd. (Shanghai, China). Fresh pork samples were obtained from a local supermarket in Harbin. ABTS [2,2′-azinobis (3-ethylbenzothiazoline-6-sulfonic acid)] and DPPH (2,2-diphenyl-1-picrylhydrazyl) were supplied by Adamas Reagent Ltd (Shanghai, China). Boric acid (H_3_BO_3_, 99.5%), magnesium oxide (MgO, 99.9%), trichloroacetic acid (C_2_HCl_3_O_2_), and trimethylamine (TMA, 30 wt% aqueous solution) were obtained from Acmec Biochemical Co. Ltd. All other reagents were purchased as analytical pure grade and utilized directly as supplied.

### 2.2. Preparation of ZIF-8-NH_2_@Rt

#### 2.2.1. Synthesis of ZIF-8-NH_2_

ZIF-8-NH_2_ was prepared through a slightly adjusted one-step synthetic route. [19]. ZIF-8-NH_2_ was precipitated by adding a methanol solution (200 mL) containing zinc nitrate hexahydrate (2.975 g) and a binary ligand mixture (6.24 g 2-methylimidazole; 0.532 g 2-aminobenzimidazole) under vigorous magnetic stirring for 1.5 h at room temperature. After centrifugation at 10,000 rpm for 15 min, the precipitate was subjected to three ethanol wash cycles to remove excess precursors and then oven-dried under vacuum at 100 °C for 24 h to afford the final ZIF-8-NH_2_ product.

#### 2.2.2. Fabrication of ZIF-8-NH_2_@Rt Composite

The synthesized ZIF-8-NH_2_ powder was dispersed in a Rt-rich methanol solution at a 1:1 ratio. The mixture was continuously stirred at 40 °C for 3 h to promote molecular encapsulation. After the reaction, the system was held at 4 °C for 12 h. Subsequently, the composite was isolated by centrifugation at 10,000 rpm for 15 min, followed by three successive washes with methanol to remove any unbound Rt residues. The quantitative analysis of rutin loading was conducted using UV-Vis spectroscopy at 361 nm, based on a standard calibration curve (y = 0.0306x − 0.0179, R^2^ = 0.99936) established for rutin. The encapsulation efficiency (EE) was defined as the percentage ratio of rutin successfully encapsulated to the total initial rutin mass. Drug loading content (DLC) was calculated as the percentage ratio of encapsulated rutin mass to the total dry mass of the ZIF-8-NH_2_@Rt nanoparticles. The DLC was measured at 15.92%, while the EE reached 31.84%.

### 2.3. Preparation of the Multifunctional Film

The multifunctional films were prepared using a solution-casting method [20]. Briefly, PVA (0.75 g, 2.5% *w*/*v*) and GG (1.5 g, 2.5% *w*/*v*) were separately dissolved in deionized water at 90 °C under magnetic stirring. After complete dissolution, the two solutions were mixed, and glycerol (30 wt% of total solids) was added as a plasticizer under continuous stirring for 3 h. Subsequently, PSPA solution (1.5 wt%, 10 mL) and ZIF-8-NH_2_@Rt dispersion (0.75 wt%, 10 mL) were incorporated into the GP matrix and homogenized at 500 rpm for 3 h. The resulting film-forming solution was degassed by ultrasonication (40 kHz, 30 min), cast onto acrylic plates (14 × 14 cm), and dried at 60 °C for 12 h to obtain the ZIF-8-NH_2_@Rt/PSPA/GP films. Control films (GP, PSPA/GP, and ZIF-8-NH_2_@Rt/GP) were prepared using the same procedure with corresponding component adjustments. The optimal ratio of PSPA to ZIF-8-NH_2_@Rt was determined based on the TMA colorimetric response of the films.

### 2.4. Characterization

#### 2.4.1. FTIR

The functional groups of ZIF-8-NH_2_@Rt were analyzed using a FTIR (Nicolet L120-000B, Thermo Fisher Scientific, Waltham, MA, USA). The powder was directly applied to the ATR crystal, and spectra were subsequently collected between 500 and 4000 cm^−1^ to assess the material’s structural characteristics. In addition, FTIR spectra of GP, PSPA/GP, ZIF-8-NH_2_@Rt/GP, and ZIF-8-NH_2_@Rt/PSPA/GP films were collected under the same conditions by directly placing the film samples onto the ATR crystal surface.

#### 2.4.2. XRD

The phase composition of ZIF-8, ZIF-8-NH_2_, and ZIF-8-NH_2_@Rt samples was analyzed using a X-ray diffractometer (D/max-2600/PC, Jeol, Tokyo, Japan). The powdered samples were evenly spread into the sample holder, flattened, and compacted for measurement. The scanning range was set from 5° to 80° (2θ) to determine the crystalline phases and structural integrity of the materials.

#### 2.4.3. TEM

Sample morphology was characterized by using a transmission electron microscope (HT-7800, Hitachi, Tokyo, Japan). Digital image processing and particle size measurements were performed with ImageJ 1.54 g software, where 50 nanoparticles were analyzed to determine size distribution.

#### 2.4.4. UV Spectroscopy and Color Rendering Performance Analysis of PSPA

The characteristic peaks of the PSPA solutions across a pH range of 3 to 12 were analyzed using a UV-Vis spectrophotometer (UV-2600, Shimadzu, Tokyo, Japan), and the color changes in both the PSPA solutions and composite films at pH 3–12 were recorded using a smartphone.

#### 2.4.5. Effect of the ZIF-8-NH_2_@Rt/PSPA Ratio on the Colorimetric Response to TMA

The addition amounts of each component in the GP films were optimized according to the color response behavior of PSPA to TMA. TMA was employed as a model volatile amine to mimic alkaline compounds released during food deterioration and to assess the sensing capability of the films. Smartphone-captured images of the films were imported into ImageJ for subsequent extraction of the red (R), green (G), and blue (B) color channel values. The ratio of the green channel to the red channel (G/R) was selected as the colorimetric response parameter. For sensitivity evaluation, the composite film was fixed over a centrifuge tube containing 600 μL of TMA solution with varying concentrations and exposed to TMA vapor for 30 min. After exposure, the film images were recorded for subsequent color analysis. Deionized water vapor was used as the blank control. The ratio between the G/R value obtained under TMA vapor and that obtained under water vapor was expressed as ΔG/R.

#### 2.4.6. SEM

Surface and cross-sectional morphologies were investigated via a SEM (SU5000, Hitachi, Tokyo, Japan) for both top-view and fracture-surface analysis. SEM characterization was performed at an accelerating voltage of 5 kV. To improve electrical conductivity and minimize charging effects under electron irradiation, the specimens were coated with a thin gold layer prior to testing and fixed onto metallic sample holders using double-sided conductive adhesive tape.

#### 2.4.7. Film Thickness and Mechanical Property Testing

Film thickness was determined using a digital micrometer (SYNTEK, Huzhou, China). According to GB/T 6672-2001 [21], five different locations were randomly measured for each sample, and the mean value was reported in millimeters (mm). The TS and E% were evaluated using an AGS-J10 universal testing instrument. For mechanical testing, the films were prepared as rectangular strips (130 mm × 15 mm) in compliance with GB/T 1040.3-2026 [22], taking care to avoid samples with visible defects or air bubbles. Before mechanical testing, the samples were equilibrated for 12 h in a desiccator containing saturated potassium carbonate solution. During the measurement process, the initial clamp distance was maintained at 100 mm, while the tensile speed was set to 50 mm/min. Each formulation was tested in quintuplicate, and the average results were calculated. TS and E% were determined using Equations (1) and (2):

(1) $$TS=\frac{F}{A}$$

(2) $$E\%=\frac{\left(L-L_{0}\right)}{L}\times 100\%$$

where

TS: Tensile strength (MPa);

F: Maximum tensile force (N);

A: Cross-sectional area of the film (mm^2^);

E%: Elongation at break;

L: Gauge length at break (mm);

L_0_: Initial gauge length (mm).

#### 2.4.8. WCA Measurement

Surface wettability was measured using a JC2000C contact angle measurement instrument (JC2000D1, ZHONGCHEN, Shanghai. China) instrument following the GB/T 30693-2014 guidelines [23]. Film specimens were attached to glass substrates and positioned on the measurement platform. Subsequently, a 3 μL droplet of deionized water was carefully dropped onto the film surface, and the contact angle value was instantly acquired through the instrument analysis software. Measurements were conducted at five separate locations for each sample, and the mean result was calculated.

#### 2.4.9. WVP

The water vapor transmission rate (WVTR) of the films was assessed in accordance with GB/T 1037-2021 [24] utilizing a standard gravimetric cup method. Circular film specimens (50 mm diameter) were tightly sealed onto weighing bottles filled with anhydrous calcium chloride by hot-melt adhesive. The prepared bottles were transferred into a desiccator maintained with saturated sodium chloride solution at ambient temperature. The variation in bottle mass was monitored periodically throughout the experiment. All samples were analyzed three times independently, and the average values were applied for WVP determination using Equation (3):

(3) $$WVP=\frac{G\times L}{A\times t\times △P}$$

where

WVP: Water vapor permeability coefficient (g·Pa^−1^·s^−1^·m^−1^)

G: Mass change in the weighing bottle over time (g)

L: Average film thickness (m)

A: Cross-sectional area of the bottle mouth (m^2^)

t: Time interval (s)

ΔP: Vapor pressure difference between the inside and outside of the bottle (Pa).

#### 2.4.10. Antioxidant Activity

Antioxidant capacities were determined employing DPPH and ABTS assays, strictly adhering to previous reports. Briefly, film specimens with identical dimensions were immersed in 10 mL freshly prepared DPPH or ABTS working solution and mixed thoroughly using a vortex oscillator. After reacting at ambient conditions for 30 min, the absorbance values were obtained using a UV–Vis spectrophotometer at 537 nm for the DPPH assay and 734 nm for the ABTS assay. The scavenging efficiencies of free radicals were determined according to Equation (4) [25].

(4) $$Antioxidant\;activity\left(\%\right)=\frac{\left(A_{0}-A_{1}\right)}{A_{0}}\times 100\%$$

A_0_ and A_1_ are the absorbance of DPPH of the control and test film, respectively,

#### 2.4.11. Light Barrier Property

The light-shielding behavior of the films was analyzed using a UV–Vis spectrophotometer Film specimens (30 mm × 20 mm) were fixed in the sample holder, and optical spectra were collected over the wavelength range of 200–800 nm. Correspondingly, the mean transmittance within the UV-B, UV-A, and visible ranges was computed via Equations (5)–(7) [26], to characterize the ultraviolet barrier performance of the films.

(5) $$T_{UV-A}=\int_{315}^{400}T_{\lambda }d_{\lambda }/\int_{315}^{400}d_{\lambda }$$

(6) $$T_{UV-B}=\int_{280}^{315}T_{\lambda }d_{\lambda }/\int_{280}^{315}d_{\lambda }$$

(7) $$T_{Visible}=\int_{400}^{760}T_{\lambda }d_{\lambda }/\int_{400}^{760}d_{\lambda }$$

where

T_λ_ represents the transmittance of the film at wavelength λ.

T_UV−A_ denotes the average transmittance in the UV-A region (315–400 nm).

T_UV−B_ refers to the average transmittance in the UV-B region (280–315 nm).

T_Visible_ indicates the average transmittance in the visible light region (400–760 nm).

#### 2.4.12. Thermal Stability

The thermal stability characteristics of the films were probed via a TGA550 thermogravimetric analyzer (TA Instruments, New Castle, DE, USA). Under flowing N_2_ (40 mL/min), ~30 mg of the films was heated from 25 to 600 °C at a constant rate of 10 °C/min.

#### 2.4.13. Antibacterial Test

The antibacterial activities of the films against *Escherichia coli* (*E. coli*) and *Staphylococcus aureus* (*S. aureus*) were evaluated [27]. The activated bacterial strains were streaked onto solid culture media, and single colonies were transferred to liquid media and incubated overnight at 37 °C with shaking. After three activation cycles, log-phase bacteria were pelleted (4000 rpm, 10 min), washed with PBS, and subsequently diluted to a working concentration of 10^6^ CFU/mL. Fabricated as 6 mm disks with a punch cutter, the samples were sterilized under UV light for 30 min. The sterilized agar medium was cooled to 40–60 °C, and 100 μL of the bacterial working suspension was added per plate. The mixture was thoroughly mixed and poured into Petri dishes. After solidification, the sample disks were gently placed onto the surface of the bacterial plates and lightly pressed to ensure full contact. After overnight incubation at 37 °C, subsequent measurement of the inhibition zone diameters was performed.

#### 2.4.14. Cycling Stability and Stability Test for the pH-Sensing

The reusability of the ZIF-8-NH_2_@Rt/PSPA/GP films was evaluated by alternately exposing the films to TMA and acetic acid vapor at 25 °C for 30 min per cycle. After each cycle, the color changes were recorded photographically. A total of four complete alternating cycles were performed on a single film sample to demonstrate the chemical reversibility and robustness of the sensing indicator, without implying practical reuse of the film for different food packages.

To evaluate long-term storage stability, the samples were maintained in a desiccator at 25 °C and 50% relative humidity (RH) for 28 days. At 7-day intervals, samples were exposed to TMA vapor for 20 min, and the colorimetric response was documented and analyzed relative to baseline measurements.

#### 2.4.15. Monitoring of Pork Freshness by Multifunctional Film

Fifty-gram pork portions were placed in transparent containers with the film adhered to the lid interior. Following a 12 h incubation at 25 °C, Subsequent colorimetric shifts in the film were documented to correlate with the freshness status of the meat.

#### 2.4.16. Preservation Test of Pork with Multifunctional Film

Fresh pork samples were randomly assigned into two groups: a control group packaged with GP film and an experimental group packaged with ZIF-8-NH_2_@Rt/PSPA/GP film. Approximately 20 g of pork was separately wrapped with the corresponding films and stored at 25 °C for 4 days. Changes in TVB-N and pH were monitored every 24 h. The TVB-N content was analyzed with a Semi-micro Kjeldahl Method, according to GB 5009.228–2016 [28], whereas pH values were determined following GB 5009.237–2016 [29].

#### 2.4.17. Statistical Analysis

All trials were replicated thrice, with data presented as mean ± SD. One-way ANOVA (SPSS 21.0) was utilized for statistical analysis, and further distinctions among groups were evaluated via Duncan’s test (*p* < 0.05).

## 3. Results

### 3.1. Characterization and Analysis of ZIF-8-NH_2_@Rt

#### 3.1.1. FTIR Analysis of ZIF-8-NH_2_@Rt

**Figure 1:**
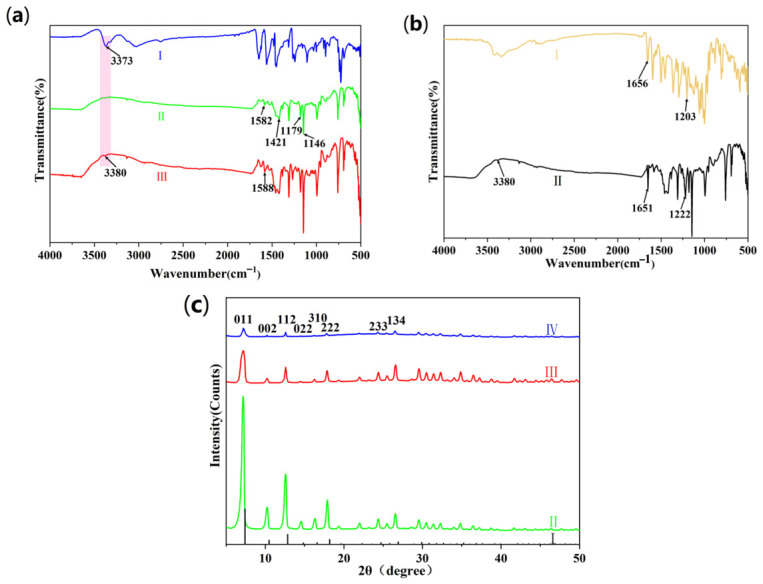
FTIR spectrum of nanoparticles: (**a**) (I) 2-aminobenzimidazole, (II) ZIF-8, (III) ZIF-8-NH_2_; (**b**) (I) Rt, (II) ZIF-8-NH_2_@Rt. XRD patterns: (**c**) (I) Simulated ZIF-8, (II) ZIF-8 (III) ZIF-8-NH_2_, (IV) ZIF-8-NH_2_@Rt.

Figure 1a,b presents the FTIR spectra of ZIF-8-NH_2_@Rt nanoparticles. For pristine ZIF-8, the characteristic absorption peaks observed at 1582, 1421, 1179, and 1146 cm^−1^ were attributed to the C=N stretching vibration of the imidazole ring, the bending vibration of –CH_3_, the C–N in-plane stretching vibration, and the imidazole ring stretching vibration, respectively [30,31,32]. After modification with 2-aminobenzimidazole, ZIF-8-NH_2_ retained the characteristic peaks of ZIF-8, while a new absorption peak appeared at 3380 cm^−1^, corresponding to the N–H stretching vibration. In addition, the characteristic peak at 1582 cm^−1^ shifted to 1588 cm^−1^, indicating successful amino functionalization of ZIF-8 [33]. For ZIF-8-NH_2_@Rt nanoparticles, the absorption peak at 1222 cm^−1^ was assigned to the C–O–C stretching vibration of Rt glycosidic bonds, while the peak at 1651 cm^−1^ corresponded to the C=O stretching vibration of Rt [34,35]. These characteristic signals confirmed the successful loading of Rt onto ZIF-8-NH_2_ and the formation of ZIF-8-NH_2_@Rt nanoparticles.

#### 3.1.2. X-Ray Diffraction Analysis of ZIF-8-NH_2_@Rt

The crystalline properties of ZIF-8, ZIF-8-NH_2_, and ZIF-8-NH_2_@Rt were analyzed by XRD. As illustrated in Figure 1c, pure ZIF-8 displayed typical diffraction peaks at 2θ values of 7.2°, 10.2°, 12.6°, 14.6°, 16.3°, 17.9°, 24.4°, and 26.6°, which were assigned to the (011), (002), (112), (022), (013), (222), (233), and (134) crystal planes, respectively [36]. Comparable diffraction patterns were also observed for ZIF-8-NH_2_ and ZIF-8-NH_2_@Rt at identical positions, suggesting that neither amino modification nor Rt encapsulation affected the original crystal framework of ZIF-8.

#### 3.1.3. TEM Analysis of ZIF-8-NH_2_@Rt

**Figure 2:**
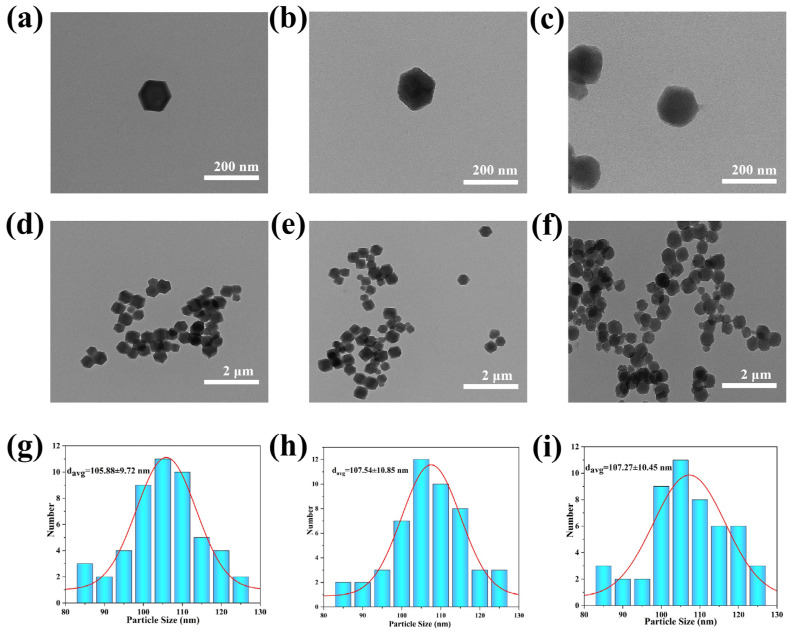
TEM images: ZIF-8 (**a**,**d**), ZIF-8-NH_2_ (**b**,**e**), ZIF-8-NH_2_@Rt (**c**,**f**). Particle size distribution histograms: ZIF-8 (**g**), ZIF-8-NH_2_ (**h**), ZIF-8-NH_2_@Rt (**i**).

Figure 2a–f display the TEM micrographs of ZIF-8, ZIF-8-NH_2_, and ZIF-8-NH_2_@Rt nanoparticles. All nanoparticles exhibited typical rhombic dodecahedral structures with distinct particle edges and without obvious aggregation, indicating favorable structural stability and dispersion behavior [37]. These results were in agreement with the XRD analysis. As shown in Figure 2g–i, the particle sizes were mainly distributed between 100 and 110 nm, demonstrating relatively uniform size distribution. Pure ZIF-8 particles were primarily distributed in the range of 80–120 nm, with the maximum proportion centered near 100 nm. After amino modification, the particle size of ZIF-8-NH_2_ increased slightly and was mainly concentrated within 90–110 nm [38]. Following Rt encapsulation, the size distribution of ZIF-8-NH_2_@Rt shifted to a relatively larger range of 100–120 nm, which may be related to the loading of Rt into the pores of ZIF-8-NH_2_ and its adsorption on the nanoparticle surface.

### 3.2. Characterization and Analysis of PSPA

**Figure 3:**
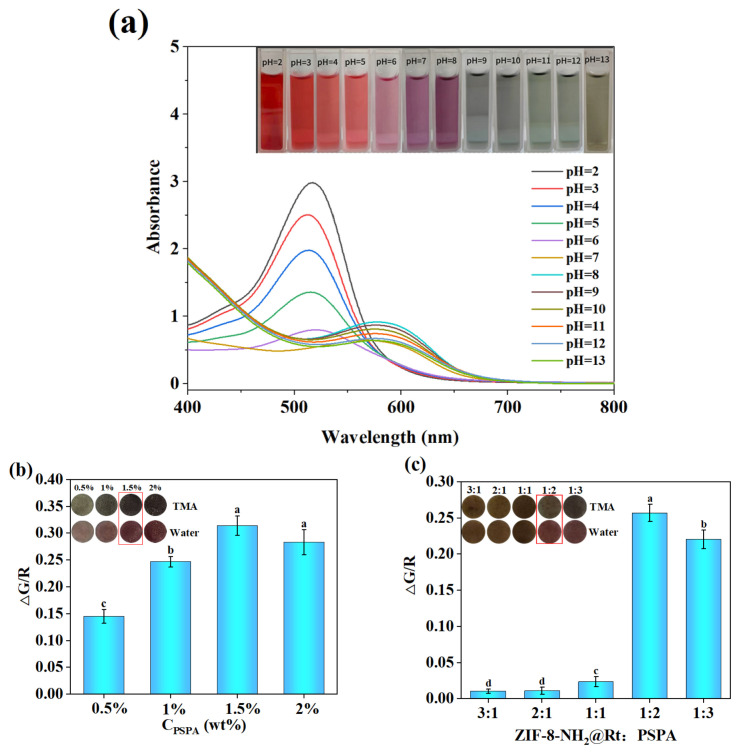
(**a**) Color change and UV-Vis spectra of PSPA at pH 2–13; (**b**) ΔG/R values at different concentrations of PSPA, (**c**) ΔG/R values at different ratios of ZIF-8-NH_2_@Rt to PSPA. different letters in the same graph indicate a statistically significant difference (*p* < 0.05).

To investigate the feasibility of PSPA as a pH-responsive indicator, its color variation under different pH environments was characterized by UV–Vis spectroscopy. As presented in Figure 3a, the PSPA solution displayed obvious color transitions as the pH increased, changing from deep red at pH 2–3 to purple at pH 6–8, and finally turning green in the alkaline range of pH 9–13 [39]. The UV–Vis spectra showed a pronounced absorption peak at 513 nm under acidic conditions (pH 2–5), which was associated with the flavylium cation structure of anthocyanins. When the pH increased to 6–7, the absorbance at 513 nm gradually declined, indicating the conversion of flavylium cations into carbinol pseudobases and partial disruption of the conjugated structure. A new absorption band appeared near 578 nm at pH 8–10, corresponding to the formation of quinoidal base species, although the absorption intensity was relatively weak because of their limited stability. Under strongly alkaline conditions (pH 11–13), the characteristic absorption peaks nearly vanished, suggesting anthocyanin degradation and the generation of chalcone-related products, which caused destruction of the conjugated system [40].

### 3.3. Optimization of the Ratio of ZIF-8-NH_2_@Rt to PSPA in GP Films

The contents of PSPA and ZIF-8-NH_2_@Rt were optimized according to the TMA colorimetric response of the films. As shown in Figure 3b, the ΔG/R value gradually increased with increasing PSPA concentration and reached its maximum at 1.5%, indicating the highest sensitivity toward TMA vapor at this level. Figure 3c further demonstrated that excessive addition of ZIF-8-NH_2_@Rt weakened the TMA response behavior of the films and caused a decline in ΔG/R values. When the mass ratio of ZIF-8-NH_2_@Rt to PSPA was adjusted to 1:2, the film exhibited the strongest response toward TMA vapor. This finding indicated that an appropriate amount of ZIF-8-NH_2_@Rt could enhance film structural stability while preserving adequate exposure of PSPA to TMA molecules. Therefore, a ZIF-8-NH_2_@Rt/PSPA mass ratio of 1:2 was selected for the following experiments.

### 3.4. Performance Analysis of ZIF-8-NH_2_@Rt/PSPA/GP Films

#### 3.4.1. SEM Analysis of ZIF-8-NH_2_@Rt/PSPA/GP Films

**Figure 4:**
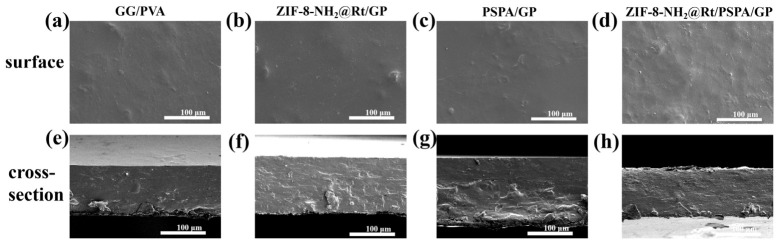
SEM images of the surface and cross-sectional morphologies of different films: (**a**,**e**) GP; (**b**,**f**) ZIF-8-NH_2_@Rt/GP; (**c**,**g**) PSPA/GP; and (**d**,**h**) ZIF-8-NH_2_@Rt/PSPA/GP.

As illustrated in Figure 4a,e, the GP film showed a compact morphology with small protuberances and wrinkles, and no signs of large particle agglomerates from undissolved guar gum. This uniformity indicates good compatibility between GG and PVA, enabling the formation of a continuous, intact film matrix. The surface wrinkles mainly result from the strong hydrogen-bonding network between the abundant hydroxyl groups in PVA chains, causing contraction and buckling during solvent evaporation [41]. Additionally, the inclusion of PSPA introduced more hydroxyl groups, increasing hydrogen-bonding density and further enhancing interfacial compatibility among all components, which effectively reduced phase separation and agglomeration [25]. As depicted in Figure 4b,d,f,h, the ZIF-8-NH_2_@Rt/PSPA/GP film exhibited a uniform distribution of ZIF-8-NH_2_@Rt nanoparticles within the GP matrix. The absence of phase separation or aggregation indicates excellent interfacial compatibility between the nanofiller and the film-forming substrate [42].

#### 3.4.2. FTIR Analysis of ZIF-8-NH_2_@Rt/PSPA/GP Films

**Figure 5:**
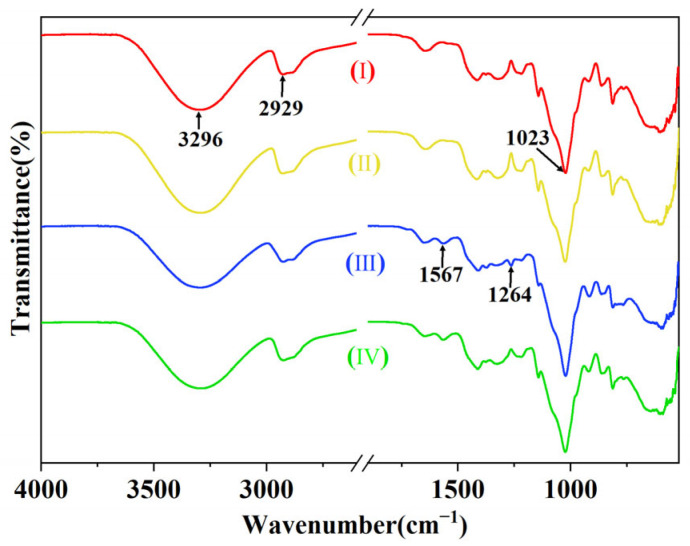
FTIR spectra of different films: (I) GP; (II) PSPA/GP; (III) ZIF-8-NH_2_@Rt/GP; and (IV) ZIF-8-NH_2_@Rt/PSPA/GP.

Figure 5 presents the FTIR spectra of the prepared films. In the neat GP film, the broad band at 3296 cm^−1^ was assigned to O–H stretching vibrations, while the absorption peaks at 2929 and 1023 cm^−1^ were related to C–H stretching and C–O–C ether bond vibrations, respectively, which are typical of the GG backbone and PVA hydroxyl groups [43,44]. After PSPA incorporation, no additional characteristic peaks appeared, indicating that no new chemical bonds were generated in the film matrix. However, the O–H absorption band became slightly broader, implying enhanced hydrogen-bonding interactions between PSPA and the GP matrix [45]. After introducing ZIF-8-NH_2_@Rt, new absorption peaks appeared at 1567 and 1264 cm^−1^, corresponding to the C=N and C–N stretching vibrations of the imidazole ring in ZIF-8, respectively [30]. In the ZIF-8-NH_2_@Rt/PSPA/GP composite film, the characteristic peaks of both ZIF-8-NH_2_@Rt and PSPA remained detectable, although partial overlap may have occurred between the ZIF-8-NH_2_@Rt peak at 1264 cm^−1^ and the PSPA peak near 1220 cm^−1^. These observations demonstrated that both ZIF-8-NH_2_@Rt and PSPA were successfully incorporated into the GP matrix.

#### 3.4.3. Mechanical Property Analysis of ZIF-8-NH_2_@Rt/PSPA/GP Films

**Figure 6:**
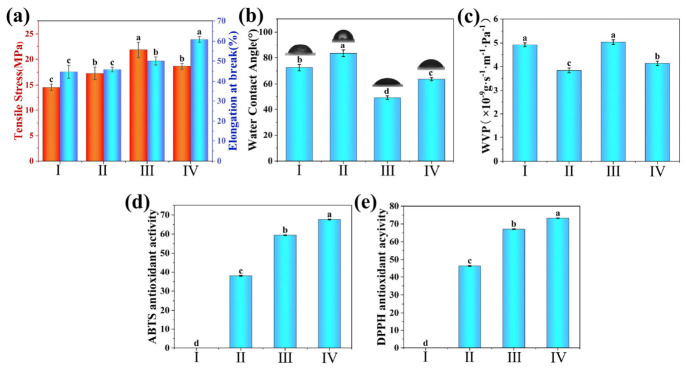
(I) GP; (II) ZIF-8-NH_2_@Rt/GP; (III) PSPA/GP; and (IV) ZIF-8-NH_2_@Rt/PSPA/GP. (**a**) TS and E% of the films. (**b**) WCA and corresponding images of the films. (**c**) WVP of the films. (**d**,**e**) Antioxidant activities of the films determined by DPPH and ABTS radical scavenging assays. Different lowercase letters within the same group indicate significant differences (*p* < 0.05).

Figure 6a presents the mechanical performance of the prepared films. Compared with the pure GP film, incorporation of ZIF-8-NH_2_@Rt increased the TS from 14.55 to 17.27 MPa, corresponding to an 18.7% improvement. This enhancement was mainly related to the reinforcing role of ZIF-8-NH_2_@Rt nanoparticles and the stronger intermolecular interactions formed between the filler and polymer matrix, especially hydrogen bonding interactions [46]. Nevertheless, the E% showed only a minor increase, indicating that the rigid nanoparticles limited polymer chain movement and decreased film flexibility, leading to a tendency toward brittle behavior [47]. In contrast, the addition of PSPA alone significantly improved both TS and E%, with increases of 50.1% and 11.8%, respectively, which was associated with the hydroxyl-rich structure of PSPA and the formation of intermolecular hydrogen-bonding networks within the GP matrix [48]. For the ZIF-8-NH_2_@Rt/PSPA/GP composite film, the TS reached 19 MPa, which remained 30.6% higher than that of the neat GP film, while the E% increased markedly from 44.72% to 61%, corresponding to a 36.6% increase. These findings suggested that the simultaneous incorporation of ZIF-8-NH_2_@Rt and PSPA effectively balanced film rigidity and flexibility, thereby improving the overall mechanical properties of the composite film.

#### 3.4.4. WCA Analysis of ZIF-8-NH_2_@Rt/PSPA/GP Films

Figure 6b illustrates the WCA values of the prepared films. Incorporation of ZIF-8-NH_2_@Rt into the GP matrix increased the WCA from 72.44° to 83.44°, indicating enhanced surface hydrophobicity. This phenomenon was primarily attributed to the intrinsic hydrophobicity of the ZIF-8-NH_2_@Rt particles, which diminished the film’s surface wettability towards water. In contrast, the PSPA/GP film showed a reduced WCA of 49.06% because the hydroxyl-rich structure of PSPA promoted interactions with water molecules through hydrogen bonding [49]. For the ZIF-8-NH_2_@Rt/PSPA/GP composite film, the WCA reached 63.69°, suggesting that ZIF-8-NH_2_@Rt partially offset the hydrophilic effect caused by PSPA. Overall, the co-incorporation of ZIF-8-NH_2_@Rt and PSPA effectively adjusted the wettability of the film surface and improved its resistance to moisture [50].

#### 3.4.5. WVP Analysis of ZIF-8-NH_2_@Rt/PSPA/GP Films

Figure 6c shows the WVP of different films. In food packaging systems, lower WVP is beneficial for limiting moisture exchange and extending product shelf life. Compared with the neat GP film, the addition of ZIF-8-NH_2_@Rt decreased the WVP from 4.922 to 3.844. This reduction was mainly related to the homogeneous dispersion of ZIF-8-NH_2_@Rt nanoparticles in the polymer network, which reduced structural micro voids and increased the diffusion path of water vapor molecules [51]. In contrast, the PSPA/GP film exhibited a higher WVP value of 5.041, mainly because the hydrophilic hydroxyl groups in PSPA promoted water adsorption and moisture migration through hydrogen bonding interactions [52]. For the ZIF-8-NH_2_@Rt/PSPA/GP composite film, the WVP decreased to 4.134, suggesting that the barrier effect of ZIF-8-NH_2_@Rt partially offset the hydrophilic nature introduced by PSPA. These findings indicate that the combined incorporation of ZIF-8-NH_2_@Rt and PSPA improved the moisture barrier property of the composite film.

#### 3.4.6. Antioxidant Activity Analysis of ZIF-8-NH_2_@Rt/PSPA/GP Films

The antioxidant potential of the films was assessed by determining the radical scavenging efficiencies against DPPH and ABTS, as illustrated in Figure 6d,e. The neat GP film showed minimal antioxidant ability because it lacked active radical-scavenging compounds, resulting in low inhibition rates toward both DPPH and ABTS radicals. After introducing ZIF-8-NH_2_@Rt, the antioxidant activity of the film increased markedly, with DPPH and ABTS scavenging efficiencies reaching 46.44% and 37.94%, respectively. This enhancement was mainly related to the flavonoid structure of Rt, which contains phenolic hydroxyl groups and conjugated double bonds that can neutralize free radicals through hydrogen donation and electron transfer. The incorporation of PSPA further improved the antioxidant performance. The PSPA/GP composite demonstrated DPPH and ABTS scavenging efficiencies of 67.11% and 59.41%, respectively, attributable to the potent radical-neutralizing capacity of the anthocyanin polyphenolic compounds [53]. Among all samples, the ZIF-8-NH_2_@Rt/PSPA/GP composite film displayed the highest antioxidant activity, with DPPH and ABTS scavenging rates of 73.27% and 67.64%, respectively. The enhanced antioxidant effect indicated a synergistic interaction between ZIF-8-NH_2_@Rt and PSPA within the composite film system [54].

#### 3.4.7. UV–Vis Light Barrier Property of ZIF-8-NH_2_@Rt/PSPA/GP Films

**Figure 7:**
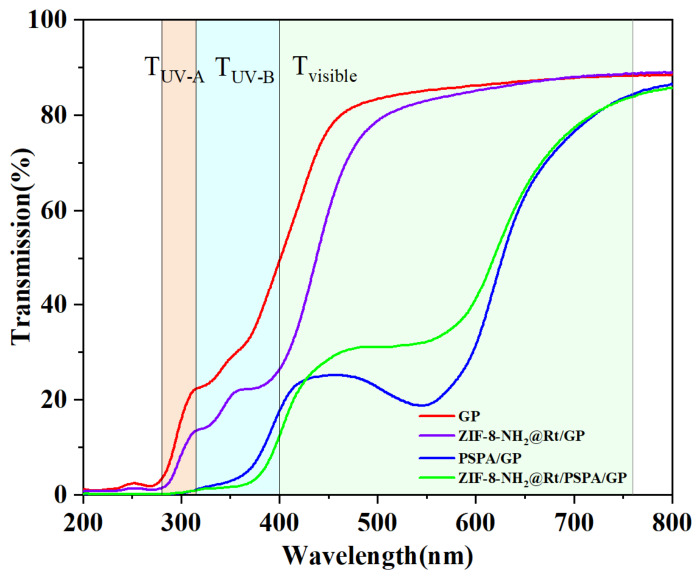
Light transmittance of guar gum/polyvinyl alcohol blend and composite films.

UV barrier efficacy was determined via full-spectrum transmittance analysis and the calculation of average transmittance values over distinct wavelength bands. As presented in Table 1 and Figure 7, the neat GP film showed weak UV-blocking ability, with average transmittances of 13.47%, 31.84%, and 82.65% in the UV-B, UV-A, and visible regions, respectively. A rapid increase in transmittance above 300 nm further indicated its limited resistance to UV light. After incorporation of ZIF-8-NH_2_@Rt, the UV-B and UV-A transmittances decreased to 7.20% and 20.05%, respectively. This enhancement was mainly related to the UV absorption property of Rt and the light-scattering effect generated by uniformly dispersed nanoparticles. For the PSPA/GP film, the UV-B and UV-A transmittances were further reduced to 0.35% and 5.57%, respectively, due to the strong ultraviolet absorption ability of anthocyanins with conjugated polyphenolic structures. Meanwhile, the obvious decrease in transmittance around 550 nm was associated with the visible-light absorption of flavylium cations in PSPA [55]. Among all samples, the ZIF-8-NH_2_@Rt/PSPA/GP film exhibited the strongest UV-shielding performance, with UV-B and UV-A transmittances of only 0.40% and 3.22%, respectively. Compared with the PSPA/GP film, the composite film displayed improved UV-A blocking capability, indicating that the combined incorporation of ZIF-8-NH_2_@Rt and PSPA effectively enhanced the UV barrier property of the film while retaining suitable visible-light transparency.

#### 3.4.8. Thermal Stability Analysis of ZIF-8-NH_2_@Rt/PSPA/GP Films

**Figure 8:**
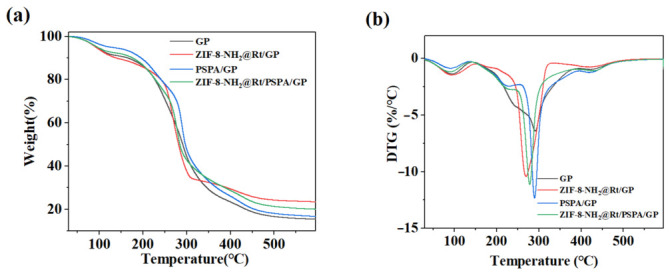
Thermogravimetric and derivative thermogravimetric curves of GP blend and composite films: TG (**a**), DTG (**b**).

The thermal behavior of the films was investigated by TG and DTG measurements. As presented in Figure 8a,b, GP, ZIF-8-NH_2_@Rt/GP, PSPA/GP, and ZIF-8-NH_2_@Rt/PSPA/GP films showed three distinct mass-loss regions during thermal decomposition. The initial mass reduction observed between 25 and 140 °C was mainly related to the evaporation of free and bound water within the film matrix [25]. The second decomposition region, ranging from 145 to 330 °C, was attributed to the degradation of small molecular substances (e.g., glycerol and Rt) together with the breakdown of polymer backbones [56]. Above 330 °C, the remaining carbonaceous components underwent oxidative degradation [57]. The most significant thermal decomposition occurred in the second stage, where the maximum degradation rate appeared at 261–297 °C. In comparison with the pure GP film, the ZIF-8-NH_2_@Rt/PSPA/GP composite film displayed a higher decomposition temperature and greater char residue, demonstrating improved thermal resistance. This enhancement could be ascribed to the intensified intermolecular interactions among ZIF-8-NH_2_@Rt, PSPA, and the GP matrix, as well as the physical barrier effect generated by uniformly dispersed nanoparticles, which limited polymer chain movement and retarded thermal degradation.

#### 3.4.9. Antibacterial Activity Analysis of ZIF-8-NH_2_@Rt/PSPA/GP Films

**Figure 9:**
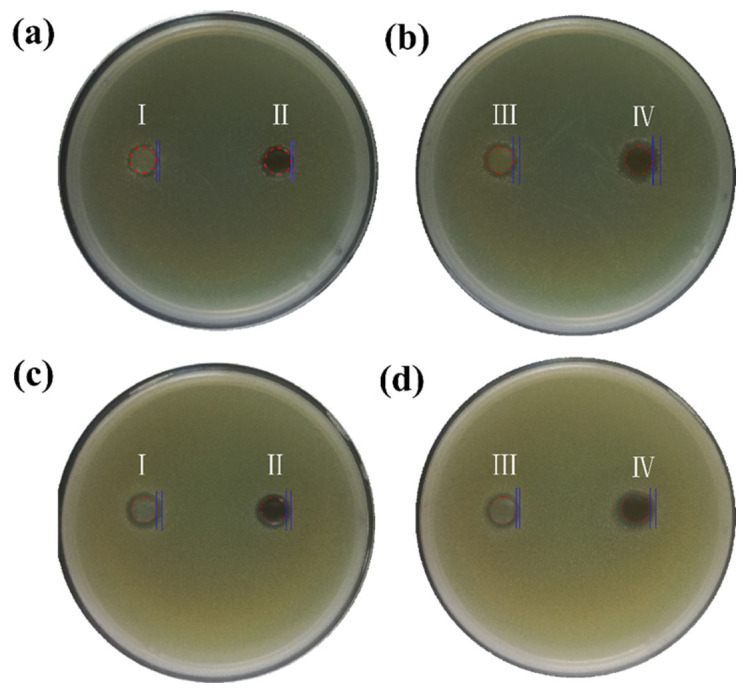
Antibacterial activities of GP and composite films against *E. coli* (**a**,**b**) and *S. aureus* (**c**,**d**): (I) GP; (II) PSPA/GP; (III) ZIF-8-NH_2_@Rt/GP; and (IV) ZIF-8-NH_2_@Rt/PSPA/GP.

The antibacterial performances of GP, ZIF-8-NH_2_@Rt/GP, PSPA/GP, and ZIF-8-NH_2_@Rt/PSPA/GP films were investigated against *E. coli* and *S. aureus*. As illustrated in Figure 9, the neat GP film showed limited inhibitory activity toward both bacterial strains. After incorporation of either ZIF-8-NH_2_@Rt or PSPA, the corresponding composite films exhibited noticeably larger inhibition zones, indicating enhanced antibacterial capability. This improvement is mainly related to the intrinsic antimicrobial activity of Rt and PSPA [34]. Among all samples, the ZIF-8-NH_2_@Rt/PSPA/GP film displayed the strongest antibacterial effect, suggesting a cooperative interaction between ZIF-8-NH_2_@Rt and PSPA in suppressing bacterial growth.

#### 3.4.10. Reusability and Stability of ZIF-8-NH_2_@Rt/PSPA/GP Films

**Figure 10:**
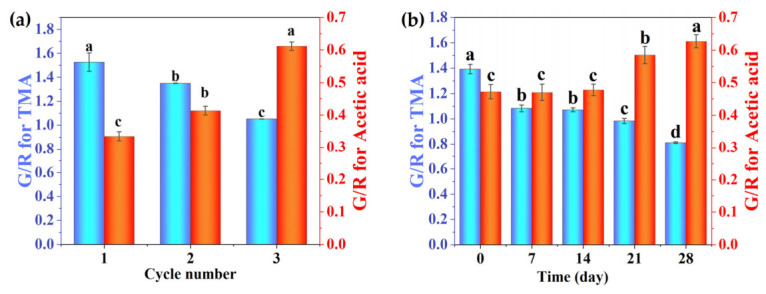
(**a**) Changes in G/R values of the ZIF-8-NH_2_@Rt/PSPA/GP film on exposure to TMA and acetic acid during cyclic tests. (**b**) Evolution of G/R values for TMA and acetic acid responses over 28 days of storage. Different letters within the same graph indicate statistically significant differences (*p* < 0.05).

The reusability and storage stability of the ZIF-8-NH_2_@Rt/PSPA/GP film are summarized in Table 2 and Table 3. Figure 10a reveals that the film’s G/R value upon TMA exposure decreased over multiple cycles, in contrast to the response to acetic acid, which increased with cycling times. Under alkaline conditions, anthocyanins are highly susceptible to oxidative ring-opening reactions (particularly at pH > 8), leading to the formation of colorless chalcone derivatives. This irreversible degradation prevents the complete restoration of the red color upon re-acidification. Consequently, the G/R response value gradually decreased with increasing cycles under basic conditions, while it exhibited a progressive increase under acidic conditions. Furthermore, the film exhibited significant chromatic drift following stimulation with TMA/acetic acid and subsequent storage for 28 days. This deterioration in performance is mainly attributed to the cumulative effect of oxidative stress and photodegradation on the unstable flavylium cation structure [58].

#### 3.4.11. Monitoring Pork Freshness Using ZIF-8-NH_2_@Rt/PSPA/GP Films

**Figure 11:**
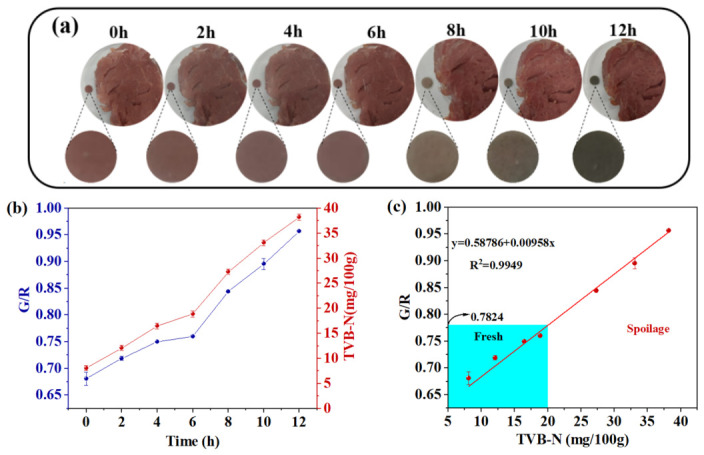
(**a**) Color change images of pork preservation film at 25 °C, (**b**) TVB-N content and G/R value curves of ZIF-8-NH_2_@Rt/PSPA/GP film, (**c**) Correlation between TVB-N content and G/R value.

**Figure 12:**
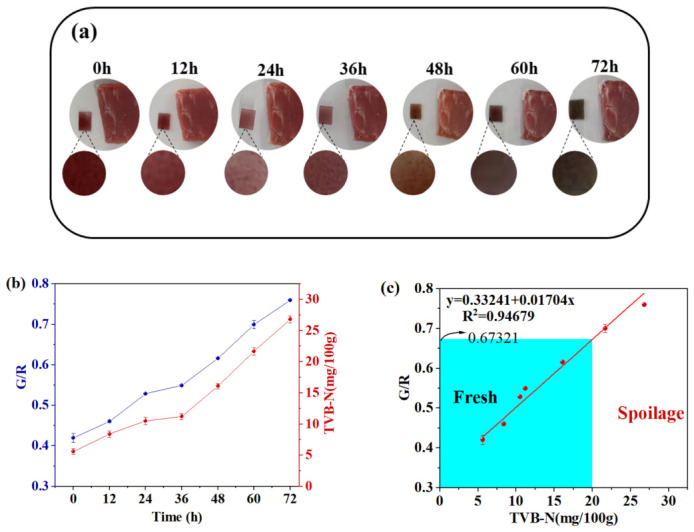
(**a**) Color change images of pork preservation film at 4 °C, (**b**) TVB-N content and G/R value curves of ZIF-8-NH_2_@Rt/PSPA/GP film, (**c**) Correlation between TVB-N content and G/R value.

Fresh pork is highly susceptible to deterioration during storage as a result of microbial growth and endogenous enzymatic degradation, which generate volatile alkaline substances [59]. The practical feasibility of the ZIF-8-NH_2_@Rt/PSPA/GP film for real-time pork freshness monitoring was evaluated under both ambient (25 °C) and refrigerated (4 °C) conditions. As shown in Figure 11a, when co-stored with pork at 25 °C for 12 h, the film exhibited a distinct color transition from pink to yellow and finally to dark green, corresponding to the continuous release of volatile alkaline compounds during spoilage. This visual evolution was quantitatively validated by Total Volatile Basic Nitrogen (TVB-N) measurements (GB 5009.228–2016) [28]. As presented in Figure 11b,c, the TVB-N content reached 27.31 mg/100 g after 8 h, exceeding the safety limit of 20 mg/100 g and indicating the pork (GB 2707–2016) [60] was unfit for consumption. To assess its applicability in cold-chain logistics, parallel experiments were conducted at 4 °C over an extended period of 72 h (Figure 12). Similarly, the film successfully tracked the spoilage process, displaying a gradual chromatic shift towards dark green in response to accumulating amines. Quantitative analysis revealed that the TVB-N value increased progressively, reaching 21.7 mg/100 g at 60 h and crossing the critical threshold. The high degree of correlation between the visual colorimetric response and the chemical TVB-N data under both temperature regimes demonstrates the robustness and reliability of the film as a smart indicator for monitoring meat freshness throughout the supply chain.

#### 3.4.12. Pork Preservation Study Using ZIF-8-NH_2_@Rt PSPA/GP Films

**Figure 13:**
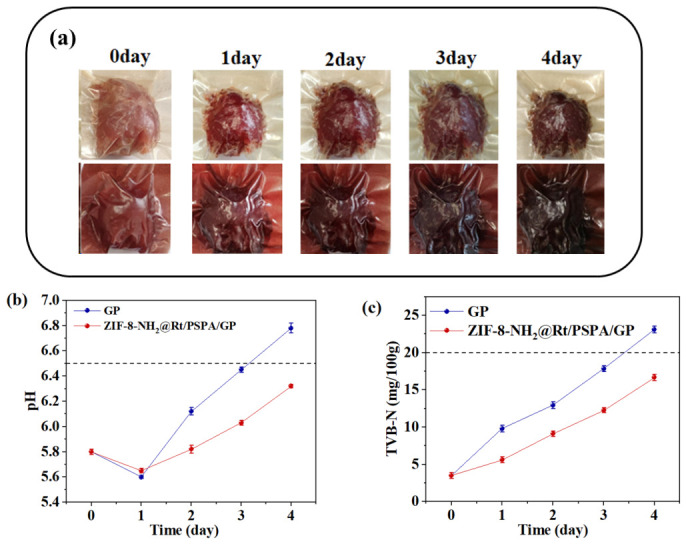
(**a**) Pork packaging experiment with at 25°C ZIF-8-NH_2_@Rt/PSPA/GP film, (**b**) pH value change curves, (**c**) TVB-N content change curves.

**Figure 14:**
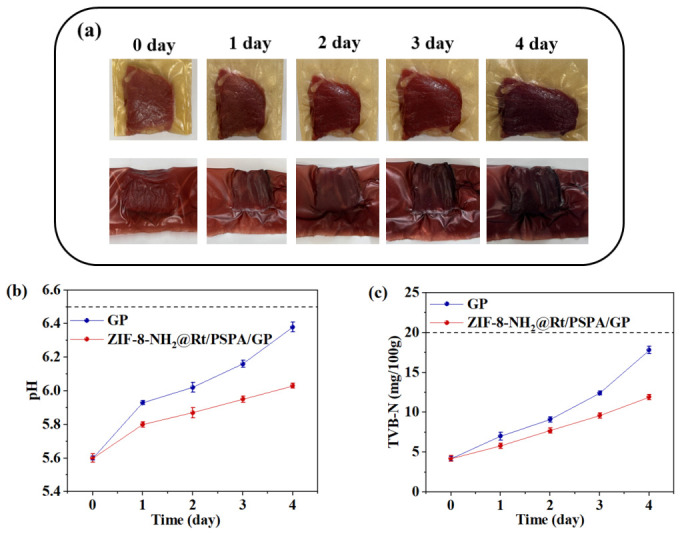
(**a**) Pork packaging experiment with at 4°C ZIF-8-NH_2_@Rt/PSPA/GP film, (**b**) pH value change curves, (**c**) TVB-N content change curves.

As demonstrated in Figure 13 and Figure 14, the composite film exhibited enhanced preservation efficacy at both 25 °C and 4 °C over 4 days. Daily monitoring confirmed that the film’s colorimetric response aligned with spoilage progression. At 25 °C, the film’s PH stabilized at 6.32 (Control: 6.78) and restricted TVB-N to 16.66 mg/100 g, preventing it from exceeding the 20 mg/100 g limit (Control: 23.1 mg/100 g). At 4 °C, similar improvements were observed, with pH maintained at 6.03 (Control: 6.38) and TVB-N at 11.9 mg/100 g (Control: 17.8 mg/100 g). These results verify that ZIF-8-NH_2_@Rt and PSPA work in tandem to inhibit microbial and oxidative spoilage, thereby validating the film’s practicality for cold-chain and ambient storage applications.

## 4. Conclusions

A novel intelligent film was developed by blending ZIF-8-NH_2_@Rt and PSPA into the GP matrix. Specifically, the rigid ZIF-8 structure reinforced the film’s strength, and PSPA concurrently increased its flexibility and break elongation. ZIF-8-NH_2_@Rt also improved the water vapor barrier property by filling internal micro voids and increasing the tortuous diffusion pathway for water molecules. Thermogravimetric analysis showed that the co-addition of ZIF-8-NH_2_@Rt and PSPA increased thermal stability owing to strong intermolecular interactions and the inherent thermal resistance of the additives. The composite films exhibited lower light transmittance, indicating enhanced UV–visible shielding. Moreover, the ZIF-8-NH_2_@Rt/PSPA/GP film could sensitively detect volatile amines with reversible color change, allowing reuse for four cycles. Based on the TVB-N correlation, G/R ratios of 0.78 (at 25 °C) and 0.67 (at 4 °C) were identified as the critical thresholds distinguishing edible from spoiled pork. The film exhibited strong antioxidant and antibacterial properties, effectively inhibiting the increase in TVB-N values.

## Figures and Tables

**Table 1:** Average light transmittance of ZIF-8-NH_2_@Rt/PSPA/GP films (%).

Sample Name	T_UV-A_	T_UV-B_	T_Visible_
GP	31.84	13.47	82.65
ZIF-8-NH_2_@Rt/GP	20.05	7.20	77.41
PSPA/GP	5.57	0.35	42.68
ZIF-8-NH_2_@Rt/PSPA/GP	3.22	0.40	47.53

**Table 2:** Reusability Test of ZIF-8-NH_2_@Rt/PSPA/GP Film.

Blank	Condition	1st Cycle	2st Cycle	3st Cycle
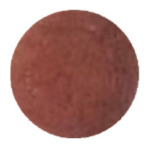	TMA	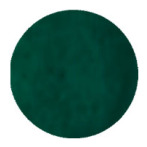	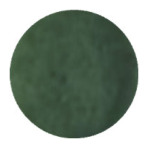	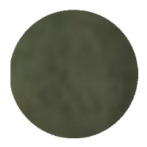
	Acetic acid	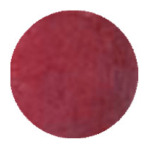	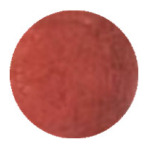	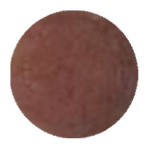

**Table 3:** Stability Test of ZIF-8-NH_2_@Rt/PSPA/GP Film.

Storage	0 Day	7 Day	14 Day	21 Day	28 Day
Temperature	25 °C	25 °C	25 °C	25 °C	25 °C
TMA	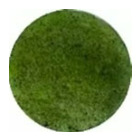	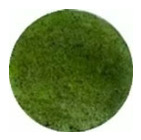	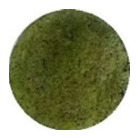	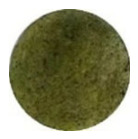	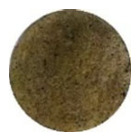
Acetic acid	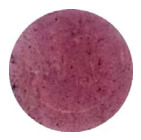	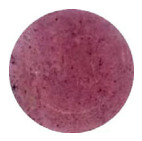	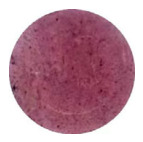	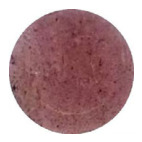	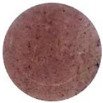

## Data Availability

The original contributions presented in this study are included in the article. Further inquiries can be directed to the corresponding author.
